# Combined Dietary Education and High-Intensity Interval Resistance Training Improve Health Outcomes in Patients with Coronary Artery Disease

**DOI:** 10.3390/ijerph191811402

**Published:** 2022-09-10

**Authors:** Pallav Deka, Jesús Blesa, Dola Pathak, Nuria Sempere-Rubio, Paula Iglesias, Lydia Micó, José Miguel Soriano, Leonie Klompstra, Elena Marques-Sule

**Affiliations:** 1 College of Nursing, Michigan State University, East Lansing, MI 48824, USA; 2 Department of Preventive Medicine, Public Health, Food Sciences, Toxicology and Legal Medicine, University of Valencia, 46010 Valencia, Spain; 3 Joint Research Unit on Endocrinology, Nutrition and Clinical Dietetics, University of Valencia-Health Research Institute La Fe, 46026 Valencia, Spain; 4 Department of Statistics and Probability, Michigan State University, East Lansing, MI 48824, USA; 5 Research Unit in Clinical Biomechanics (UBIC), Department of Physiotherapy, University of Valencia, 46010 Valencia, Spain; 6 University Clinic of Nutrition, Physical Activity and Physiotherapy, University of Valencia, 46010 Valencia, Spain; 7 Food & Health Lab, Institute of Materials Science, University of Valencia, 46980 Valencia, Spain; 8 Department of Health, Medicine and Caring Sciences, Linkoping University, 58185 Linkoping, Sweden; 9 Physiotherapy in Motion, Multispeciality Research Group (PTinMOTION), Department of Physiotherapy, University of Valencia, 46010 Valencia, Spain

**Keywords:** coronary artery disease, high-intensity exercise, resistance training, body mass index, dietary education

## Abstract

Background: Reducing cardiovascular risk through lifestyle changes that include a heart-healthy diet and regular exercise is recommended in the rehabilitation of patients with coronary artery disease (CAD). We pilot-tested the effectiveness of a dietary-education and high-intensity interval resistance training (DE–HIIRT) program on healthy food choices and associated anthropometric variables in patients with established CAD. Methods: A total of 22 participants, aged 60.0 ± 7.2 years, were enrolled in the study. Over 3 months, under the guidance and supervision of a physiotherapist, participants performed the resistance exercises 2×/week in a group setting (cohort of 11). Participants additionally attended three sessions of dietary education led by a dietician. Participants demonstrated their knowledge and understanding of dietary education by picking heart-healthy foods by reading food labels. Outcomes included change in diet (measured using the tricipital skinfold thickness Mediterranean Diet Adherence questionnaire (MEDAS-14) and the Food Consumption Frequency Questionnaire (FCFQ)) and anthropometric measurements (body composition, body circumference, and tricipital skinfold thickness). A paired t-test was performed to analyze the differences between the baseline and post-intervention results. Results: Participants significantly increased their consumption of vegetables (*p* = 0.04) and lowered their consumption of sweet snacks (*p* = 0.007), pastries (*p* = 0.02), and processed food (*p* = 0.05). Significant improvements in body mass index (*p* = 0.001), waist circumference (*p* = 0.0001), hip circumference (*p* = 0.04), and body fat (*p* = 0.0001) were also achieved. Conclusion: Making lifestyle changes that include both diet and exercise is essential in the management of CAD. The HIIRT program combined with dietary changes shows promise in achieving weight-loss goals in this population and needs to be further investigated with appropriate study designs.

## 1. Introduction

Cardiovascular diseases (CVDs) are the leading cause of death worldwide, and the numbers are expected to rise in the future [1]. Ischemic heart disease, often from coronary artery disease (CAD), accounts for the vast majority of deaths associated with CVDs [2]. Significant stenosis and/or symptomatic CAD patients often undergo either a percutaneous coronary intervention (PCI) or a coronary-artery bypass grafting (CABG) procedure to improve myocardial perfusion [3]. Post-PCI, the risk of restenosis is high (about 36%) [4], and the cost of treatment and mortality associated with it is significant [5]. As such, lifestyle modifications for diet and exercise are essential in the onset, treatment, progression, and prevention of the reoccurrence of CAD [6].

The rise in the consumption of processed food among the general population and the rise in the prevalence of CVD go hand in hand [7]. Dietary change and making healthy food choices, such following the Mediterranean diet (MedD), are essential to combatting CVD [8,9]. Additionally, the American Heart Association and the European Society of Cardiology also recommend regular exercise for improving and maintaining a healthy weight for cardiovascular health [10,11]. 

Although, traditionally, recommendations for exercise in cardiac rehabilitation have included mostly continuous moderate-intensity aerobic exercise training [12], recent studies have also investigated the use of aerobic high-intensity interval training for cardiac rehabilitation with favorable results [13]. With its effectiveness shown to be equal to or greater than conventional training [14], high-intensity aerobic exercise has been incorporated into recently published clinical guidelines as a useful alternative [15]. Moderate-intensity resistance training is effective in improving muscle strength, functional capacity, and mobility in CAD patients [16]. However, an investigation of the effects of high-intensity interval resistance training (HIIRT), specifically when combined with education on a healthy diet, in patients with established CAD is lacking.

It is now well-accepted that, to reduce cardiovascular risk, lifestyle changes in both diet and exercise are important. Newer forms of exercise, such as HIIRT, should be investigated for their potential health benefits. Therefore, the purpose of this pilot study was to compare the effectiveness of combining dietary education (on MedD) and a high-intensity interval resistance training program (DE–HIIRT) on the making of healthy food choices and anthropometric variables (body composition and body circumferences) in patients with CAD. 

## 2. Methods

### 2.1. Study Design and Sample

This study was a 3-month-long, single-group, experimental trial (ClinicalTrials.gov Identifier: NCT03796234). Participants were recruited from a rehabilitation clinic in Valencia. Patients were eligible if: (i) they were above 18 years of age; (ii) they were diagnosed with CAD and overweight (body mass index (BMI) > 25); (iii) at least 6 months had elapsed between the diagnosis of CAD and the beginning of the study; (iv) they were physically and cognitively able to complete the evaluations and the program; and (v) they had clearance from a cardiologist to participate in the program. The exclusion criteria were: (i) a diagnosis of structural heart disease, atrial fibrillation/flutter, ischemic, or non-ischemic heart failure; (ii) they were currently engaged in performing structured exercise; (iii) they had physical or psychiatric conditions that prevented the normal carrying out of evaluations and the program; and (iv) they had a positive Bruce treadmill stress test. Our sample size was adequate for a pilot study [17].

The study was conducted according to the principles of the Declaration of Helsinki, and the protocol was approved by the Human Research Ethics Committee (H1476979767902) at the University of Valencia. 

**Intervention:** Our physiotherapist- and dietician-supported face-to-face intervention was grounded in Bandura’s Self-Efficacy Theory, which suggests that competence and confidence in performing a task can be enhanced through the accomplishment of the task, receiving positive feedback, vicarious experience, and the symbolic desensitization of emotions (such as anxiety associated with high-intensity exercise) [18,19]. We recorded attendance at the group-specific interventions as described below. 

**HIIRT Exercise:** The group-based HIIRT program was delivered by a cardiac physiotherapist with more than 10 years of experience with CAD patients, twice a week, for 3 months. The program involved 2 days/week of high-intensity interval training (24 sessions in total), which is adequate in patients with CAD [20,21]. Sessions were divided into warm-up (10 min), exercise (30 min), and cool-down (10 min). Blood pressure was checked at the beginning and at the end of each session. The warm-up period included light walking with a series of eight flexibility exercises (isotonic hip and knee flexo-extension, shoulder and elbow flexo-extension), followed by six self-stretches. The exercise part included lower- and upper-limb resistance exercises (hip/knee flexion-extension, hip abduction, shoulder flexion-extension, elbow flexion-extension, triceps flexion-extension, and shoulder abduction); each exercise was performed for 1 min. The intensity was maintained at 60–70% of 1 repetition maximum (RM) throughout the intervention period. This was followed by a 1-min active recovery break, which included 0.5-kilogram (kg) dumbbell exercises (shoulder flexion-extension, elbow flexion-extension, triceps flexion-extension, and walking). Exercises were performed in a circuit, and we incorporated walking in the low-intensity phase to provide a break from continuously performing resistance exercises. The circuit allowed participants to complete two bouts of each exercise. We used a conservative approach during the high-intensity phase, with the amount of resistance at 60–70% of 1 RM, but we encouraged the participants to perform more repetitions in the 1-min period as the weeks progressed. Also, for safety, we monitored heart rates with a heart-rate monitor during exercise. Cool-down included the same exercises and self-stretching as the warm-up. 

**Dietary Education**: The dietary education program focused on the MedD, which involves the use of whole grains, fruits, vegetables, legumes, and fish, with extra-virgin olive oil as the main fat, in addition to the moderate consumption of meat, products high in saturated and trans fats, sugars, etc. [7] Dietary education included 3 group-based, 2-h long dietary-education workshops within the first month of enrollment. The education was delivered by the same dietician, who has more than 5 years of experience in working with cardiac patients. Each workshop was followed by a question-and-answer session. Workshops included a participative oral presentation and worksheets to encourage the active participation of the audience. Broadly, education was divided into three categories with associated topics. The topics covered were as follow: **(a) *Healthy eating and food myths*:** basic concepts about healthy eating; benefits of healthy eating; the relationship between CAD and a healthy diet; nutrients in a healthy diet; and food myths; **(b) *Organizing daily diet:*** implementing healthy eating in daily life; weekly organization and making shopping lists; reviewing nutrients and their importance; organizing the pantry; learning how to draw up a weekly menu and how to stylize dishes; and ***(c)*** ***Interpreting nutrition labeling***: the importance of labeling; the components of a label; reading labels; and a labeling workshop for picking healthy foods by reading labels. Additionally, at the end of each workshop, participants were given a dossier summarizing the information taught during the lessons.

### 2.2. Primary Outcome

**Healthy food choice:** to assess the difference in the consumption of individual food items, the **Food Consumption Frequency Questionnaire (FCFQ)** was used. The questionnaire registers the number of times per week, or the number of times per month, that a series of foods is consumed to determine the foods that the individual consumes most frequently during a week. The list of foods that are part of the questionnaire is made up of those that are most commonly consumed by the population, with a total of 45 items (yogurt, fish, fruits, etc.). It also includes a table, called the Item Portion Weight Table, in which the weight of each item’s portion is listed so as to facilitate the completion of the questionnaire. We used the Spanish version of the questionnaire, the reliability and validity of which has been established [22].

### 2.3. Secondary Outcome

Anthropometric parameters included:

**(i) Body composition:** an OMRON HBF-500INT bioelectrical impedance meter was used to calculate body fat (%). A SECA scale was used to measure weight (kg) and height (meters (m)).

**(ii) Body circumferences:** a flexible-steel CESCORF measuring tape was used with an accuracy of 1 mm. Specifically, we assessed waist circumference (centimeters (cm)), hip circumference (cm), calf circumference (cm), and arm circumference (cm). 

### 2.4. Procedure

All patients with a diagnosis of CAD who visited the cardiology clinic were evaluated for inclusion criteria by a physiotherapist. Participants signed an informed consent prior to enrollment. At baseline, participants provided demographic and clinical information, completed the questionnaires, and performed a maximal-effort treadmill stress test using the Bruce protocol to capture maximal heart rate and establish their ability to perform high-intensity exercise with anginal symptoms or changes in an electrocardiogram [23]. The anthropometric assessment was carried out according to the application of the International Standards for Anthropometric Assessment guidelines [24]. All participants were provided with one session of individualized acquaintance with the resistance exercises after enrollment and baseline evaluation. The physiotherapist demonstrated the exercises and asked participants to demonstrate each exercise in turn. The resistance for each participant was individualized. For each group, participants were divided into 2 cohorts, with 7–8 participants in each cohort. Exercising together in a group allowed the participants to observe others so as to build self-efficacy vicariously. The physiotherapist provided encouragement and positive feedback during the exercises. In the following 3 months, participants received 24 sessions of physiotherapist-guided training in the HIIRT program. Additionally, participants received dietary education. Post-intervention data were collected at the end of 3 months. Patients were contacted by telephone by a research team member to make an appointment for the follow-up assessments, which took place in the clinic. 

### 2.5. Data Analysis

Descriptive data of demographic and clinical characteristics are shown as means (standard deviations) or frequencies, as appropriate. The Shapiro–Wilk test was used to evaluate the normality of the data. A paired t-test was performed to determine differences across the two time points. Only food items from the FCFQ that were found to be significant were reported. The effect size of the intervention was analyzed. The level of significance was set at α = 0.05. All statistical analyses were performed using IBM’s SPSS software (Version 25.0; IBM Corp., Armonk, NY, USA).

## 3. Results

A total of 32 subjects were found to be eligible, with 10 participants refusing to participate for personal reasons. We enrolled 22 participants in the study. Attendance was >85% at the prescribed group-specific intervention sessions. All participants completed the baseline and post-intervention data collection and were included in the final analysis. 

Of the sample, 18 subjects were men (81.8%), with a mean age of 61.1 ± 6.5 years. With a mean BMI of 30.7 ± 4.5 kg/m^2^, class I obesity (BMI between 30.0–34.9 kg/m^2^) was observed at baseline, with 93% of participants being overweight or obese. While no participant currently smoked, 15 participants reported to being former smokers. At enrollment, all patients stated they were physically active at home, but none were engaged in any form of active exercise. All participants had undergone a prior percutaneous coronary intervention procedure post-CAD diagnosis. Table 1 shows the demographic and clinical characteristics of the participants.

At 3 months, we found significant improvement in both the primary and secondary outcomes. Uponn analyzing the FCFQ data, over time, the participants significantly improved their consumption of vegetables (*p* = 0.04) and decreased their consumption of sweet snacks (*p* = 0.007), pastries (0.027), and processed food (*p* = 0.05). Among the anthropometric measures, a significant difference was seen in BMI (29.0 ± 3.2; *p* = 0.001), waist circumference (*p* = 0.0001), hip circumference (*p* = 0.04), and body fat (*p* = 0.0001). The mean weight loss was 4.4 kg (80.4 ± 11.9 kg). Table 2 shows the changes in anthropometric outcome measures that were found significant.

A post-hoc analysis was performed to calculate the effect size of our intervention using the primary outcome (BMI). A small effect size (Cohen’s d) of 0.42 was found.

## 4. Discussion

Our pilot study shows that a program combined with dietary education and HIIRT provides important therapeutic benefits. We found significant improvements in food choices and anthropometric measures, pre-and post-intervention. Participants significantly increased their consumption of vegetables and decreased their intake of sugary items and processed food. To our knowledge, this is the first study that evaluates the effectiveness of a combined program of HIIRT exercise and dietary education.

Our study highlights that CAD patients who are looking to lose weight will benefit by making lifestyle changes that combine HIIRT with a healthy diet that includes a higher consumption of fruits and vegetables and lower consumption of high-caloric and processed foods. These findings are consistent with previous studies that have found that dietary education can improve dietary diversity, improving quality of life in patients with heart disease [25,26]. The positive changes in diet and improvement in the choice of food consumption is also reflected in the participants achieving significant improvement in anthropometric measures, namely BMI, waist circumference, hip circumference, and body fat. These improvements are consistent with the theoretical framework that an increase in muscle mass from resistance training will increase basal metabolic rate, which will aid in increasing total caloric expenditure and in lowering body fat [27]. Increases in strength can aid in improving physical activity status, and in the presence of a lower caloric diet, weight loss can be enhanced through resistance training [27]. In our sample, we found the mean weight to decrease by 4.40 kg over 3 months. The observed weight loss is consistent with the recommendation of healthy weight loss of 0.45–0.90 kg/week for reducing the cardiovascular risk profile and reducing clinical events in overweight and obese individuals [28,29].

The MedD has been shown to have an important impact on cardiovascular diseases [30]. Our results show that dietary education improves the consumption of healthy foods in CAD. These results are consistent with previous studies, which have found that an educational group strategy did improve the adoption of a MedD in patients without CVD [31] and with hypertension [32]. An important component of our dietary education intervention was to allow participants to demonstrate their understanding of the educational content by arranging a workshop for picking healthy foods by reading labels. Such a method of active participation may be a better approach to dietary education. Previous interventions seem to have mostly focused on traditional approaches of providing only lecture-based education, individually or in groups [33]. Regarding anthropometric parameters, we found significant improvements in BMI, waist circumference, hip circumference, and total body fat. This is consistent with previous studies on patients with CAD [34] and other populations [35] that have demonstrated the positive impact of nutritional education on improving anthropometric parameters [6,10].

A systematic review and meta-analysis on aerobic high-intensity interval exercise training in patients with CAD found 11 studies that reported on improvements in aerobic capacity [14]. Nevertheless, there is no previous study evaluating the effectiveness of high-intensity interval training including resistance exercises combined with dietary education in CAD patients; thus, we highlight the novelty of our DE–HIIRT program. It would appear that aerobic high-intensity interval-training exercise may be as effective as either moderate-intensity interval training or continuous aerobic-exercise training, both of which are more commonly used in patients with CAD [14]. As the intensity of exercise increases, a smaller number of days or duration of exercise is recommended. For example, exercise guidelines recommend 150 min/week of moderate-intensity aerobic exercise vs. 75 min/week of high-intensity aerobic exercise. Additionally, only 2–3 days/week of resistance exercise is currently recommended. As the exercises performed in the study were high-intensity, and with no prior study to back up, we restricted the HIIRT exercises to 2 days/week. Considering that the reoccurrence of coronary artery stenosis in patients with CAD with percutaneous coronary intervention is high [4], and adherence to exercise and cardiac rehabilitation in this population remains low [36,37,38], it is imperative to test the efficacy of alternate forms of exercise programs.

The DE–HIIRT protocol allowed for a hands-on demonstration of educational content, as well as an economical and accessible alternative in resistance exercises that can be easily performed at home using dumbbells and resistance bands. We did not have any dropouts in 3 months, which indicates that a 2-day/week HIIRT program coupled with diet changes is acceptable in this population and can address the issues of low adherence that are generally seen with exercise programs. However, improvement in self-efficacy, long-term adherence to such a program, and the maintenance of the weight loss seen in our study need to be evaluated with longitudinal study designs. It is to be noted that resistance training can help reduce cardiovascular risk factors even without significant weight loss [27]. However, future studies should measure physiological parameters such as peak oxygen consumption, ejection fraction, muscle strength, and microvascular changes, as well as evaluating the quality-of-life outcomes of such an intervention.

Due to a small sample size that consisted of a larger proportion of males than females, the results should be interpreted with caution for gender-specific effects, and more studies using a similar protocol should be conducted with larger sample sizes. However, this limitation is consistent with pilot studies testing the feasibility of a novel intervention. The HIIRT was performed in a supervised setting, and the long-term safety and sustainability of performing such exercise by CAD patients in an unsupervised setting should also be investigated.

Baseline physical activity level was subjectively measured during enrolment, which may not be representative of actual levels of exercise. We also did not measure physical activity during the study period to determine the effect of our intervention on activity levels and the impact of improved physical activity on overall weight loss. Neither knowledge nor attitudes about a healthy diet, nor the kind of diet patterns participants had already adopted before enrolment, were evaluated. As such, the degree of change needed to follow a MedD is not known. Future studies should evaluate this at baseline and, if needed, incorporate innovative intervention designs [39] to address participants’ needs. Future studies should take this into account as it is an important factor in caloric expenditure. Smoking is an important cardiovascular risk factor. Our study also included a sample that consisted of CAD patients who were currently nonsmokers. The impact of such a program on participants who smoke may be different and needs to be investigated.

Scarce literature about combined protocols including HIIRT and dietary education made it difficult to compare our results with other studies. Importantly, this pilot study utilized a quasi-experimental design, and we acknowledge the confounding effect of the two different interventions on study outcomes. Future studies should be performed to establish the efficacy of each intervention with a randomized controlled trial design. Our study can serve as a starting point for investigating this line of work. These limitations should be taken into account in designing future trials.

## 5. Conclusions

Based on our findings, we conclude that our combined protocol, based on dietary education and HIIRT, improves the cardiovascular risk profile in CAD patients. Such programs should be further evaluated by researchers as a treatment of choice in the management of CAD patients. Healthcare providers should strive to provide education on heart-healthy diets, making good food choices, and incorporating resistance exercise for reducing cardiovascular risk factors.

## Figures and Tables

**Table 1:** Demographic and clinical characteristics of the participants.

	n = 22
Age (mean ± SD)	60.0 ± 7.2
Sex (frequency/%)	
Males	18/81.8
Females	4/18.2
Marital status (frequency/%)	
Single	1/4.5
Married	19/86.4
Divorced	0/0
Widowed	2/9.1
Education level (frequency/%)	
Primary	8/36.4
Secondary	9/40.9
University	5/22.7
Diabetes (frequency/%)	
Type I	1/4.5
Type II	3/13.6
Smoking (frequency/%)	
Smoker	0/0
Former smoker	15/68.2
Hypertension (frequency/%)	12/54.5
Dyslipidemia (frequency/%)	18/81.8
Family history (frequency/%)	10/35.5
Weight (kg)	84.8 ± 14.6
Height (cm)	166.1 ± 8.8

Data are expressed as means (standard deviations) or as frequencies/percentages. % = percentages; SD standard deviation; kg = kilogram; cm = centimeter.

**Table 2:** Changes in anthropometric outcomes across time.

Measure	Time	Mean	SD	95% CI		*p*-Value (2-sided)
				LB	UB	
**BMI**	**1**	**30.8**	**4.3**	28.9	32.9	0.001
	2	29.1	3.2	27.2	30.9	
**WC**	1	101.2	10.2	96.3	106.1	0.0001
	2	96.5	8.6	91.6	101.5	
**HC**	1	104.3	7.2	101.1	107.5	0.43
	2	103.9	6.8	100.9	107.0	
**BF**	1	32.1	6.4	29.2	35.1	0.0001
	2	27.8	5.5	25.3	30.3	

SD = standard deviation; CI = confidence interval; LB = lower branch; UB = upper branch; BMI = body mass index; WC = waist circumference; HC = hip circumference; BF = body fat.

## Data Availability

Not applicable.
